# Supplementary material for: Coherent acoustic control of a single silicon vacancy spin in diamond
Source: Nat Commun. 2020 Jan 10;11:193. doi: 10.1038/s41467-019-13822-x (PMC6954199; doi:10.1038/s41467-019-13822-x)
Supplement: Supplementary file 1 — Supplementary Information [file 41467_2019_13822_MOESM1_ESM.pdf]

Supplementary Information for: Coherent Acoustic Control of a Single Silicon Vacancy Spin in Diamond

Maity et al.

Supplementary Note 1: Coordinate systems

We use x, y, z to refer to the coordinate system of the devices. The axis normal to the diamond surface is denoted by z, which is the [001] lattice direction. The surface acoustic waves (SAWs) are launched along x, or the [110] lattice direction. The y direction is given by the cross product of z and x, and is along $[\bar{1}10]$.

The local coordinate system of the SiVs is referred to as X, Y, Z. The C_3 symmetry axis of the SiV, which is the line joining the two missing carbon atoms, is denoted by Z. This can be along any of the 4 equivalent $\langle 111 \rangle$ lattice directions. Because the SAWs are launched along [110], the 4 SiV axis directions are divided into two classes based on their orientation with respect to the [110] direction. "Longitudinal" SiVs have Z along [111] or $[11\bar{1}]$, and "transverse" SiVs have Z along $[1\bar{1}1]$ or $[\bar{1}11]$. The response of the two SiVs along both directions within each class is expected to be the same due to mirror symmetry of the SAWs about the $(\bar{1}10)$ plane. The X and Y directions are indicated in Supplementary Fig. 1.

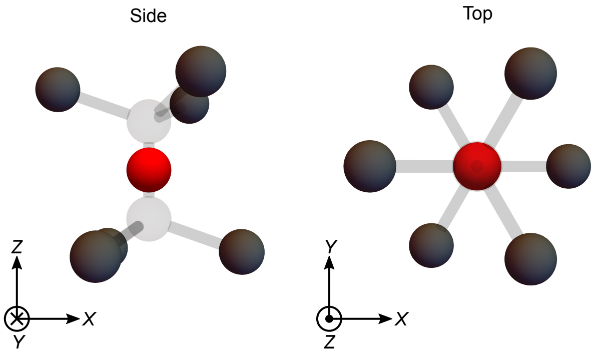

Supplementary Figure 1. Local coordinate system of the SiV.

Supplementary Note 2: Time domain characterization of SAW devices

To perform time-domain characterization, half of the electrical input to the device is sent to a real-time oscilloscope (Tektronix MSO71604C) by way of a 50-50 RF power splitter. One of the IDTs in a pair is excited with the AWG, and the signal from the other IDT is also connected to the oscilloscope. Short electrical pulses are generated with the AWG and both the AWG pulse and the transmitted electrical pulse from the SAW are characterized (Supplementary Fig. 2). The ringing of the input signal is caused by the RF amplifier. The transmitted output signal has two main features:

1. The earliest feature is due to direct electrical crosstalk between the input and output ports. It has approximately the same length as the input pulse. This is likely from the wire bonds to the input and output transducers, because these GHz-frequency electrical signals have a relatively large wavelength in air. The delay between the input signal and the crosstalk signal is due to the longer path via RF cables to the device inside the cryostat and back.

2. Next, there is the feature from the transduced SAW pulse. This ramps up and down, indicating the build-up and decay of the acoustic pulse that is expected from the non-zero length of the transducer.

The delay between the electrical crosstalk and the first acoustic response indicates a time of about 21 ns for the SAW pulse to travel between the transducers. Given that the separation between the transducers is approximately 175 μm, the SAW velocity is approximately 8.3 km s^{-1} which agrees with simulations. The non-zero length of the SAW transducer imposes a lower bound on the shortest acoustic pulses it can generate, which is about 13 ns. As the length of the microwave pulse from the AWG is increased, the length of the acoustic pulse remains approximately constant while its amplitude increases. For pulses longer than 13 ns, the acoustic pulse reaches its maximum amplitude and its length starts to increase, as expected.

Because of the minimum duration of the SAW pulses, we use a minimum delay of 10 ns between $\pi/2$ pulses for the Ramsey interferometry measurement. At this delay, the acoustic pulses overlap neglegibly (Supplementary Fig. 3).

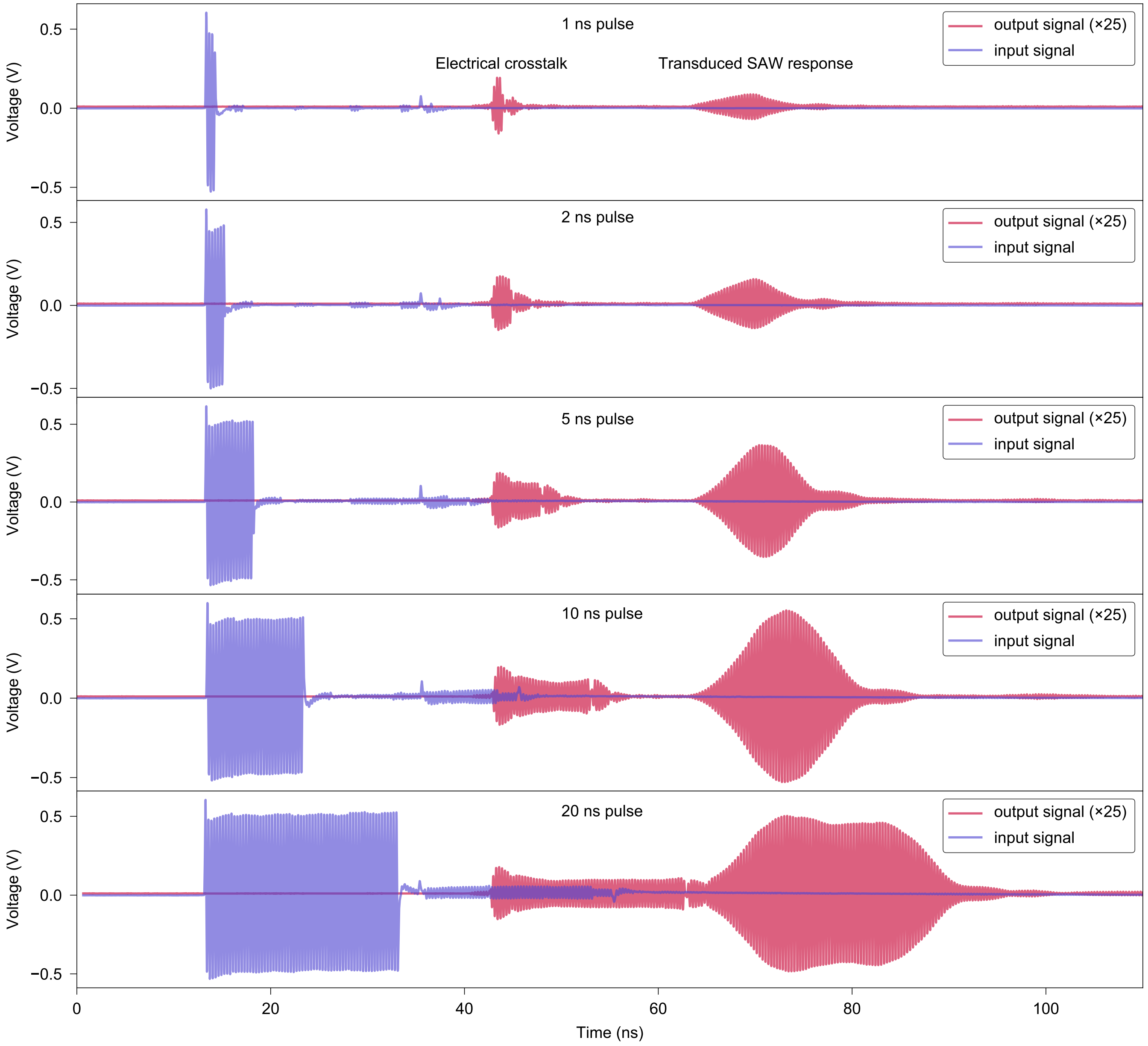

Supplementary Figure 2. Time domain characterization of SAW devices with short electrical pulses. The frequency of the pulses was set to 3.37 GHz for optimal transducer response.

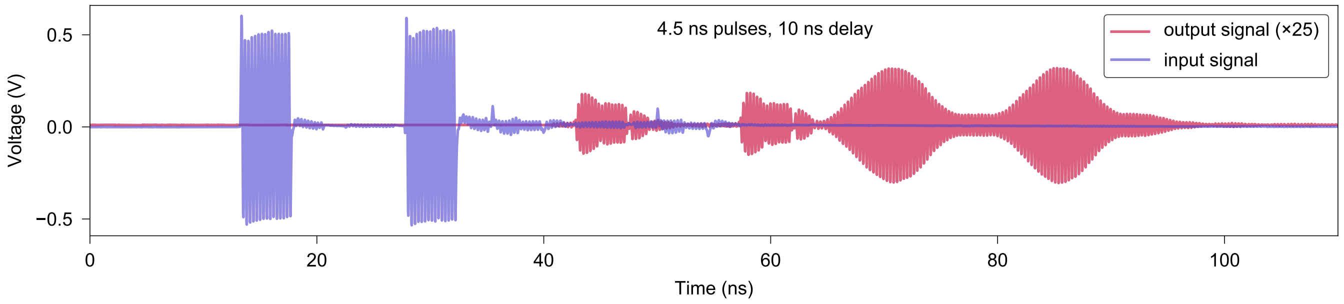

Supplementary Figure 3. Time domain chracterization of SAW devices using two 3.38 GHz, 4.5 ns $\pi/2$ pulses separated by 10 ns.

Supplementary Note 3: Calibration of optical and acoustic pulse timing

The fluorescence intensity from an SiV under optical excitation can vary in the presence of an acoustic pulse. This is observed when the laser frequency is resonant with a transition to a virtual state generated by the acoustic pulse, as explained in Supplementary Note 6. We explore this interaction by performing pulsed measurements of an SiV under a magnetic field, using different frequencies of optical and acoustic pulses, as shown in Supplementary Fig. 4. The SiV spin is initialized and read out using laser pulses, similar to the experiments in the main text, and an acoustic pulse is applied during the initialization pulse. In (a), the laser is detuned by $f = 3.34$ GHz from the C3 transition, and the SAW frequency is set to the detuning f. The laser frequency is not resonant with any of the other transitions. Hence we notice very low fluorescence intensity during the optical pulse, but there is a large increase in counts when the acoustic pulse is applied, due to the photon-phonon transition being resonant with C3. In (b), the laser is resonant with the C1 transition, and the SAW frequency is set to the difference between the C1 and C3 transitions, equivalent to the splitting between the lower ground states $|e_{g+}\downarrow\rangle$ and $|e_{g-}\uparrow\rangle$ of the SiV. There is an initial increase in the fluorescence due to the optical pulse, followed by the exponential decay as the spin population is optically pumped to $|e_{g-}\uparrow\rangle$. When the acoustic pulse is applied, the photon-phonon transition is resonant with C3, thereby leading to a large increase of fluorescence.

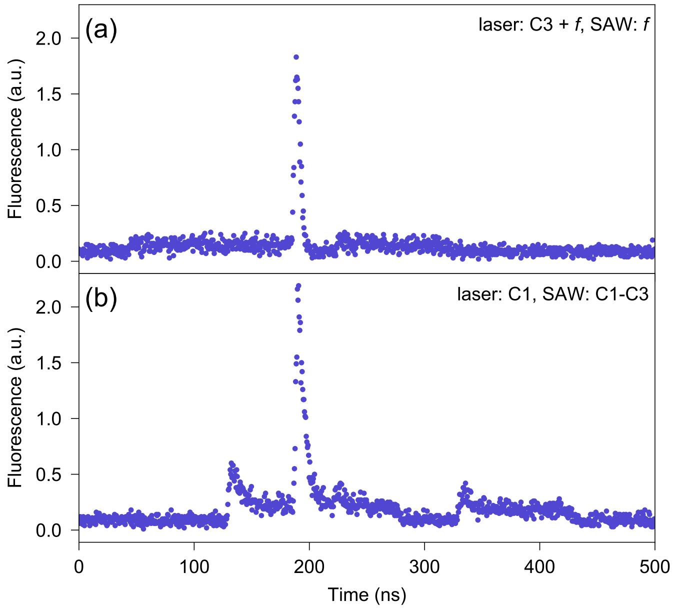

Supplementary Figure 4. Pulsed optical and acoustic measurements demonstrating A_{1g} strain response of the SiV. (a) The laser is detuned from the C3 transition by the same amount as the SAW frequency f, and is not resonant with any of the other transitions. (b) The laser is resonant with the C1 transition, and the SAW frequency is equal to the difference between the C1 and C3 transitions.

This response has practical significance for our experiment, as it allows the identification of the time of arrival of the acoustic pulse at the SiV. This is used to measure the velocity of the acoustic pulse on the diamond surface, and to establish that it is indeed the acoustic pulse that is responsible for controlling the SiV spin. To do this, we choose a different SiV, one located close ($\sim$ few μm) to one of the transducers (i.e. the "right" transducer). We repeat the measurement from Supplementary Fig. 4(b), first generating the acoustic pulse by exciting the right transducer. Then we swap the RF cables connected to the IDTs, in order to switch the roles of the left and right transducers. We repeat the measurement, this time by exciting the left transducer. The resulting time-resolved fluorescence (Supplementary Fig. 5) indicates a time difference of about 13.3 ns between the arrival of the acoustic pulses from the right and left at the SiV. Because we swap the RF cables for the second measurement, the time difference from different lengths of RF cables to the left and right ports of the cryostat is eliminated. There is still a small difference in the lengths of the wires inside the cryostat, however this difference is on the order of a few cm and is thus neglegible. Therefore the measured time difference is close to the time taken for the SAW pulse to travel between the left and right transducer. Given that the separation between transducers is 175 μm, we estimate a velocity of 13.1 km s^{-1} for the SAW. While

the estimate of the SAW velocity from the SiV fluorescence measurement and electrical measurements differ, they are both close (within 25%) of the simulated value (10.3 km s^{-1}). This indicates that these are indeed acoustic pulses driving the SiV, and not microwave radiation that might originate from the patterned electrodes (e.g. the IDTs).

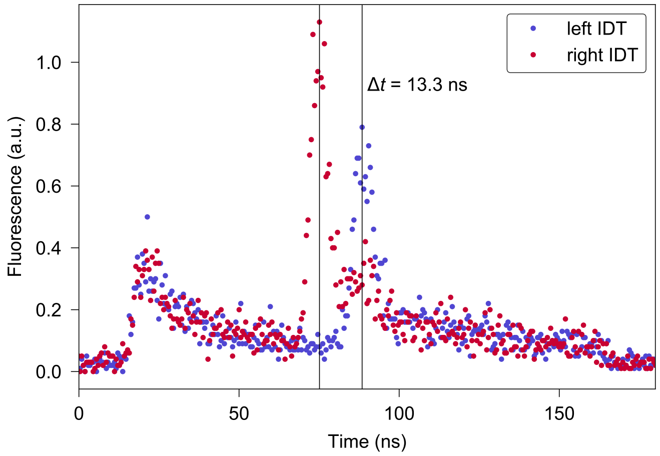

Supplementary Figure 5. Measurement of SAW velocity using SiV fluorescence.

Supplementary Note 4: Fitting of Rabi oscillation data

As mentioned in the main text, we use a simple two-level model (exponentially damped sinusoid) to fit the Rabi oscillation data. This model fits the data fairly well for low acoustic powers, but begins to show deviations at higher powers (Supplementary Fig. 6). Fitting the data using a master equation model which considers all 4 levels in the SiV ground state, similar to the approach in [1], does not provide any significant improvement over the simple model. We believe that the distortions in the oscillations may be due to reflections of the acoustic pulses from the opposing IDTs. Fabrication of IDTs individually, instead of in pairs, may provide an answer to this question.

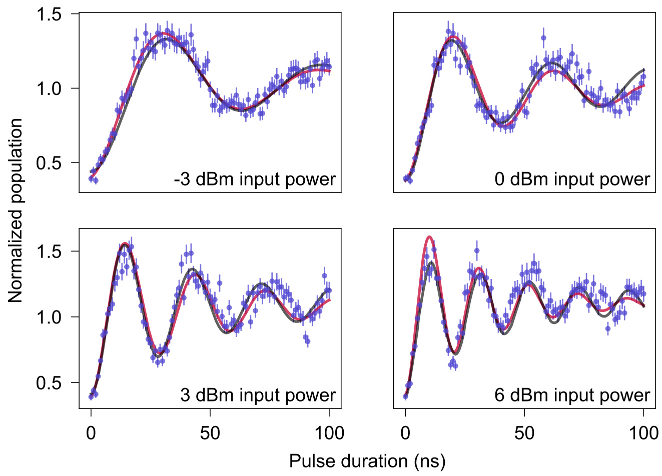

Supplementary Figure 6. Rabi oscillation measurement data for all 4 input powers. The red and black curves show the results of the two-level model and the four-level master equation model, respectively. The error bars represent the standard deviation of the normalized population.

Constant	Value	Unit	Reference
$\lambda_{\text{SO},g}$	45	GHz	[2]
$\lambda_{\text{SO},e}$	257	GHz	[2]
γ_S	28	GHz/T	[2]
γ_L	14	GHz/T	[2]
q	0.1	1	[2]
$t_{\parallel,e} - t_{\parallel,g}$	-1.7	PHz/strain	[3]
$t_{\perp,e} - t_{\perp,g}$	0.078	PHz/strain	[3]
d_g	1.3	PHz/strain	[3]
d_e	1.8	PHz/strain	[3]
f_g	-1.7	PHz/strain	[3]
f_e	-3.4	PHz/strain	[3]

Supplementary Table 1. Measured constants of the SiV Hamiltonian

Supplementary Note 5: SiV Hamiltonian

The SiV has four states in both the ground and excited state manifolds, given by the direct product of the orbital $\{|e_X\rangle, |e_Y\rangle\}$ and spin $\{|\uparrow\rangle, |\downarrow\rangle\}$ bases. For the purposes of our experiments, the SiV Hamiltonian can be expressed as a sum of spin-orbit, Zeeman, and strain terms [2, 3]. The constants appearing in these expressions have two sets of values for the ground and excited states. The spin-orbit term is given by

$$\mathcal{H}_{\text{SO}} = -\frac{\lambda_{\text{SO}}}{2} L_Z S_Z \tag{1}$$

$$= -\frac{\lambda_{\text{SO}}}{2} \begin{bmatrix} 0 & i \\ -i & 0 \end{bmatrix} \otimes \begin{bmatrix} 1 & 0 \\ 0 & -1 \end{bmatrix} \tag{2}$$

in which λ_{SO} is the spin-orbit coupling. The Zeeman term is given by

$$\mathcal{H}_{\text{Z}} = q\gamma_L L_Z B_Z + \gamma_S \vec{S} \cdot \vec{B} \tag{3}$$

$$= q\gamma_L B_Z \begin{bmatrix} 0 & i \\ -i & 0 \end{bmatrix} \otimes \mathcal{I}_2 \tag{4}$$

$$+ \frac{\gamma_S}{2} \mathcal{I}_2 \otimes \begin{bmatrix} B_Z & B_X - iB_Y \\ B_X + iB_Y & -B_Z \end{bmatrix} \tag{5}$$

in which $\gamma_L = \mu_B/\hbar, \gamma_S = 2\mu_B/\hbar$ are the orbital and spin gyromagnetic ratiosrespectively, and q is the orbital quenching factor. The strain Hamiltonian is given by

$$\mathcal{H}_{\text{strain}} = \begin{bmatrix} \alpha - \beta & \gamma \\ \gamma & \alpha + \beta \end{bmatrix} \otimes \mathcal{I}_2 \tag{6}$$

in which α, β, γ are symmetry-adjusted linear combinations of strain tensor components and are given by

$$\alpha = t_\perp(\epsilon_{XX} + \epsilon_{YY}) + t_\parallel \epsilon_{ZZ}, \tag{7}$$

$$\beta = d(\epsilon_{XX} - \epsilon_{YY}) + f\epsilon_{ZX}, \tag{8}$$

$$\gamma = -2d\epsilon_{XY} + f\epsilon_{YZ}. \tag{9}$$

Hence α consists of strain components belonging to the A_{1g} representation of the D_{3d} symmetry group and β, γ consist of the E_{gX}, E_{gY} representation components. The measured values of the constants associated with each of the terms in the Hamiltonian have been reported previously and are summarized in Supplementary Table 1.

Supplementary Note 6: Strain response of the SiV

In the coordinate system of the devices (Fig. 2(a) of the main text), the SAW mostly has components in the x and z directions, with x being the direction of propagation. Hence the components of the strain involving y can be neglected. From our simulations, we notice that the components ϵ_{xx} and ϵ_{zz} are in phase while ϵ_{xz} is $\pi/2$ out of

phase. Hence the strain tensor of the SAW in device coordinates may be expressed as

$$\epsilon_{\text{device}} = \begin{bmatrix} \epsilon_{xx} & 0 & -i\epsilon_{xz} \\ 0 & 0 & 0 \\ -i\epsilon_{xz} & 0 & \epsilon_{zz} \end{bmatrix}. \tag{10}$$

The SiV responds differently to strain components belonging to the A_{1g} and E_g representations. The A_{1g} strain components ϵ_{ZZ} and $\epsilon_{XX}+\epsilon_{YY}$ (in SiV coordinates) shift the energies of the SiV electronic states, while the E_g strain components $\epsilon_{XX}-\epsilon_{YY}$, ϵ_{XZ}, ϵ_{XY} and ϵ_{YZ} mix the electronic orbitals [3]. While the E_g strain response is responsible for the acoustic spin control, the A_{1g} response is also of practical significance to this experiment, as mentioned in Supplementary Note 3.

A. A_{1g} strain response

When the SiV is subjected to an oscillating A_{1g} strain field, the oscillating energy shift of the electronic states causes frequency modulation of the energy levels, generating virtual states from interaction with an integer number of phonons. These virtual states are revealed as sidebands in the resonant excitation spectrum under continuous-wave acoustic driving (Supplementary Fig. 7). As the acoustic power is increased, more sidebands are observed. We observe two sidebands using 6 dBm of microwave input power applied to the transducer, demonstrating photon-phonon transitions with up to two phonons.

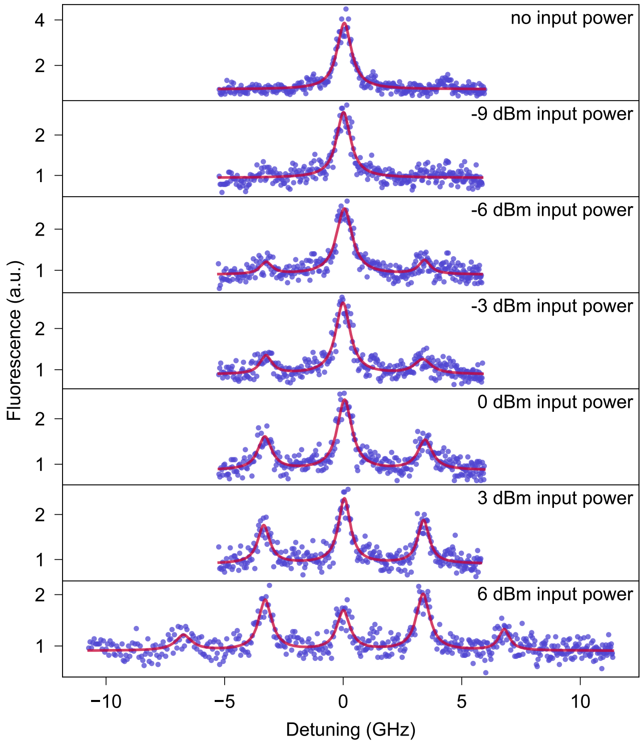

Supplementary Figure 7. Frequency modulation-induced sidebands of the SiV C-transition at zero magnetic field with varying acoustic powers.

Since these measurements are performed at zero magnetic field, the SiV can be described by just the spin-orbit and strain Hamiltonian terms:

$$\mathcal{H} = \mathcal{H}_{\text{SO}} + \mathcal{H}_{\text{strain}} \tag{11}$$

$$= -\frac{\lambda_{\text{SO}}}{2} \begin{bmatrix} 0 & i \\ -i & 0 \end{bmatrix} \otimes \begin{bmatrix} 1 & 0 \\ 0 & -1 \end{bmatrix} + \begin{bmatrix} \alpha - \beta & \gamma \\ \gamma & \alpha + \beta \end{bmatrix} \otimes \mathcal{I}_2 \tag{12}$$

resulting in the energies

$$E_\pm = \alpha \pm \sqrt{\left(\frac{\lambda_{\mathrm{SO}}}{2}\right)^2 + \beta^2 + \gamma^2}. \tag{13}$$

The instantaneous quantities α, β, γ are given by the sum of a static term like α_0 arising from pre-strain, and a time-dependent term like $\widetilde{\alpha}\sin(\omega t)$ from the oscillating SAW drive. For the SiV used in our measurements, for which $\beta, \gamma \ll \lambda_{\mathrm{SO}}$, the instantaneous energy shift of the C-transition is given by

$$\Delta E_C = \alpha_e - \alpha_g. \tag{14}$$

As this is linear in strain, the time-dependent part of the energy shift is independent of any existing pre-strain and is given by

$$\Delta E_C(t) = (\widetilde{\alpha}_e - \widetilde{\alpha}_g)\sin(\omega t). \tag{15}$$

This energy shift induces virtual states that couple to the lower excited state, with relative quasi-energies given by integer multiples of the drive frequency,

$$\Delta E_{C,k} = k\omega \tag{16}$$

and populations

$$P_k \propto \left| J_k \left(\frac{\widetilde{\alpha}_e - \widetilde{\alpha}_g}{\omega} \right) \right|^2 \tag{17}$$

in which J_k are Bessel functions of the first kind.

SAW power estimation. Assuming that the SiV is located at the focus of the Gaussian SAW beam, finite element simulation allows us to determine the value of $\Delta\widetilde{\alpha} = \widetilde{\alpha}_e - \widetilde{\alpha}_g$, for a given SAW power P, from the strain tensor amplitude ϵ. However, in our experiments, the power at the location of the SiV is lower than the input microwave power by an efficiency factor η. Therefore, the quantity $\Delta\widetilde{\alpha}$ for an input power P_{in} is given by

$$\Delta\widetilde{\alpha} = \Delta\widetilde{\alpha}_{\mathrm{sim}} \sqrt{\frac{\eta P_{\mathrm{in}}}{P_{\mathrm{sim}}}} \tag{18}$$

where $\Delta\widetilde{\alpha}_{\mathrm{sim}}, P_{\mathrm{sim}}$ are the values obtained from simulation. By using this relation, we fit the observed relative fluorescence intensities to the populations P_k for different input powers, while varying the parameter η. The measured and fitted relative fluorescence intensities are shown in Supplementary Fig. 8. We determine that the SiV is oriented longitudinally with respect to the SAW propagation direction, and estimate $\eta = 3.4 \times 10^{-4}$. The various factors contributing to η are discussed further in Supplementary Note 7.

B. E_g strain response

Our calculations are performed in the basis of the eigenstates of the sum of the spin-orbit and axial Zeeman Hamiltonians, as we work in a non-zero magnetic field, in which we treat the strain and transverse magnetic field as perturbations. The initial Hamiltonian is given by

$$\begin{aligned} \mathcal{H}_0 &= \mathcal{H}_{\mathrm{SO}} + \mathcal{H}_{\mathrm{Z}} && (19)\\ &\approx -\frac{\lambda_{\mathrm{SO}}}{2}\begin{bmatrix} 0 & i \\ -i & 0 \end{bmatrix} \otimes \begin{bmatrix} 1 & 0 \\ 0 & -1 \end{bmatrix} + \frac{\gamma_S}{2}\mathcal{I}_2 \otimes \begin{bmatrix} B_Z & 0 \\ 0 & -B_Z \end{bmatrix} && (20)\\ &= \frac{1}{2}\begin{bmatrix} \gamma_S B_Z & 0 & -\lambda_{\mathrm{SO}} i & 0 \\ 0 & -\gamma_S B_Z & 0 & \lambda_{\mathrm{SO}} i \\ \lambda_{\mathrm{SO}} i & 0 & \gamma_S B_Z & 0 \\ 0 & -\lambda_{\mathrm{SO}} i & 0 & -\gamma_S B_Z \end{bmatrix} && (21) \end{aligned}$$

in which we have neglected the effect of the magnetic field on the orbitals, due to the small orbital quenching factor $q = 0.1$ [2]. The eigenstates are given by $\{|e_{g+}\uparrow\rangle, |e_{g-}\downarrow\rangle, |e_{g-}\uparrow\rangle, |e_{g+}\downarrow\rangle\}$, in which $|e_{g\pm}\rangle = |e_{gX}\rangle \pm i\,|e_{gY}\rangle$, with energies given by $(\pm\lambda_{\mathrm{SO}} \pm \gamma_S B_Z)/2$. The off-axis magnetic field term is

$$\mathcal{H}_{\mathrm{Z,off}} = \frac{\gamma_S}{2}\mathcal{I}_2 \otimes \begin{bmatrix} 0 & B_X - iB_Y \\ B_X + iB_Y & 0 \end{bmatrix} \tag{22}$$

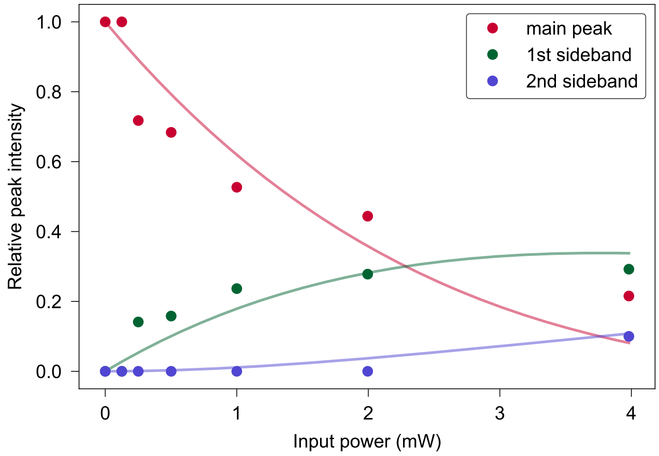

Supplementary Figure 8. Measured and fitted relative fluorescence intensities from Supplementary Fig. 7, used for estimation of the power efficiency factor.

which in the eigenstate basis is

$$\mathcal{H}_{\mathrm{Z,off}} = \frac{\gamma_S}{2}\begin{bmatrix} 0 & 0 & 0 & B_- \\ 0 & 0 & B_+ & 0 \\ 0 & B_- & 0 & 0 \\ B_+ & 0 & 0 & 0 \end{bmatrix} \tag{23}$$

in which $B_\pm = B_X \pm iB_Y$. This perturbs the eigenstates as

$$|e_{g+}\downarrow\rangle' \approx |e_{g+}\downarrow\rangle + \frac{\gamma_S B_+}{2\lambda_{\mathrm{SO}}}|e_{g+}\uparrow\rangle\,, \tag{24}$$

$$|e_{g-}\uparrow\rangle' \approx |e_{g-}\uparrow\rangle + \frac{\gamma_S B_-}{2\lambda_{\mathrm{SO}}}|e_{g-}\downarrow\rangle\,. \tag{25}$$

The strain term in the eigenstate basis is

$$\mathcal{H}_{\mathrm{st}} = \begin{bmatrix} \alpha & 0 & -\beta - i\gamma & 0 \\ 0 & \alpha & 0 & -\beta + i\gamma \\ -\beta + i\gamma & 0 & \alpha & 0 \\ 0 & -\beta - i\gamma & 0 & \alpha \end{bmatrix} \tag{26}$$

which introduces a coupling between $|e_{g+}\downarrow\rangle$ and $|e_{g-}\uparrow\rangle$, given as

$$\Omega = \langle e_{g+}\downarrow'|\mathcal{H}_{\mathrm{st}}|e_{g-}\uparrow'\rangle \tag{27}$$

$$= \frac{\gamma_S}{2\lambda_{\mathrm{SO}}}\left(B_-(-\beta - i\gamma) + B_-(-\beta - i\gamma)\right) \tag{28}$$

$$= -\frac{\gamma_S(\beta + i\gamma)(B_X - iB_Y)}{\lambda_{\mathrm{SO}}} \tag{29}$$

which is the generalized Rabi frequency when the oscillating strain field is resonant with the $|e_{g+}\downarrow\rangle \leftrightarrow |e_{g-}\uparrow\rangle$ transition, and the instantaneous quantities β, γ are replaced by the amplitudes $\widetilde{\beta}, \widetilde{\gamma}$. The magnitude of Ω is equal to the measured Rabi frequency.

SAW power estimation. Similar to the case of the A_{1g} strain response, the Rabi frequency for a given SAW power is estimated from simulation, and the measured Rabi frequencies are given by

$$\Omega = \Omega_{\mathrm{sim}}\sqrt{\frac{\eta P_{\mathrm{in}}}{P_{\mathrm{sim}}}}. \tag{30}$$

The linear fit of Ω against $\sqrt{P_{\mathrm{in}}}$ (Fig. 4(a) in the main text) gives $\eta = 3.7\times10^{-5}$. This differs from the efficiency factor estimated from the A_{1g} strain response by a factor of 9.2, and potential reasons for this discrepancy are discussed in Supplementary Note 7.

Supplementary Note 7: SAW power efficiency

The power efficiency factors estimated from the A_{1g} strain response and E_g response (Rabi oscillations) are given by

$$\eta_{A_{1g}} = 3.4 \times 10^{-4}, \quad (31)$$
$$\eta_{E_g} = 3.7 \times 10^{-5} \quad (32)$$

as mentioned in Supplementary Note 6. These factors can be expressed as a product of several individual efficiencies:

$$\eta_{A_{1g}} = \eta_{\text{device}}, \quad (33)$$
$$\eta_{E_g} = \eta_{\text{device}} \times \eta_{\text{magnetic}} \times \eta_{\text{pre-strain}}, \quad (34)$$
$$\eta_{\text{device}} = \eta_{\text{input}} \times \eta_{\text{propagation}} \times \eta_{\text{alignment}}. \quad (35)$$

The interpretation of each of these factors is explained below.

1. η_{input} is the conversion efficiency of the input microwaves to SAW by the transducer. It is estimated to be about 8.8×10^{-2} from the electrical S_{11} measurement (Fig. 2(b) in the main text).

2. $\eta_{\text{propagation}}$ is due to the propagation losses of the SAWs travelling from the IDT to the SiV. Assuming that the conversion efficiencies for microwaves to SAW and SAW to microwaves are the same, this factor is estimated to be about 3.2×10^{-1} which we infer from the electrical S_{11} and S_{21} measurements.

3. $\eta_{\text{alignment}}$ is due to the spatial misalignment between the SiV and the focus of the Gaussian SAW beam. Due to the large separation between the two IDTs in our device, it is not possible to determine the exact location of the focus during experiments, as both IDTs cannot be simultaneously imaged within one optical field of our confocal microscope. To identify the focal point, we first locate one IDT and then translate the optical field to where we expect the focus to be, after which we search for SiVs. It is possible to be misaligned by up to a few μm. The effect of this misalignment on the efficiency factor is shown in Supplementary Fig. 9. Assuming a worst-case misalignment of 5 μm, this factor can be as low as 3.9×10^{-3}. In addition, misalignment along y can also introduce yy, xy, and zy components in the strain tensor, which changes the relations between $\Delta\alpha$, Ω, and P. We do not attempt to evaluate these effects in detail.

4. η_{magnetic}. In our simulations, we assume that the magnetic field is oriented along the [001] direction. However, the direction of the magnetic field lines of a permanent magnet vary significantly depending on the spatial location. This introduces some uncertainty in the magnetic field components, which can vary the Rabi frequency.

5. $\eta_{\text{pre-strain}}$. The Rabi frequency from the E_g strain response is significantly reduced by any existing pre-strain [3]. However, the A_{1g} strain response is robust against pre-strain. This factor accounts for the difference.

With the estimates for the efficiency factors mentioned above, η_{device} varies between 2.8×10^{-2} and 1.1×10^{-4} depending on the misalignment. Therefore, the measured efficiency factor $\eta_{\text{device}} = 3.4 \times 10^{-4}$ is within reasonable limits. Additionally, $\eta_{E_g}/\eta_{A_{1g}} = \eta_{\text{magnetic}} \times \eta_{\text{pre-strain}} = 0.11$ indicates that $\eta_{A_{1g}}$ and η_{E_g} are reasonably consistent, suggesting good agreement of our experimental results with the theory of the SiV strain response [3], up to certain effects such as the precision of the magnetic field direction and uncontrollable pre-strain.

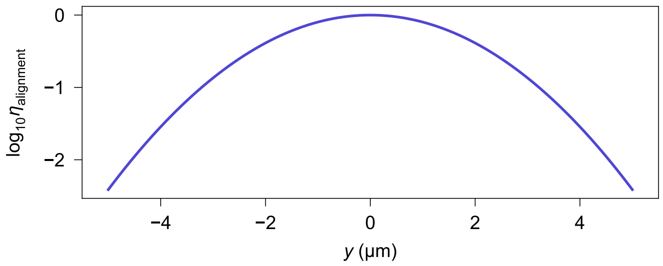

Supplementary Figure 9. Alignment efficiency factor as the location of the SiV is varied along the y-axis. The focus of the Gaussian SAW beam is at $y = 0$.

Supplementary References

[1] Benjamin Pingault, David-Dominik Jarausch, Christian Hepp, Lina Klintberg, Jonas N. Becker, Matthew Markham, Christoph Becher, and Mete Atatüre, "Coherent control of the silicon-vacancy spin in diamond," Nature Communications **8**, 15579 (2017).

[2] Christian Hepp, Tina Müller, Victor Waselowski, Jonas N. Becker, Benjamin Pingault, Hadwig Sternschulte, Doris Steinmüller-Nethl, Adam Gali, Jeronimo R. Maze, Mete Atatüre, and Christoph Becher, "Electronic Structure of the Silicon Vacancy Color Center in Diamond," Physical Review Letters **112**, 036405 (2014).

[3] Srujan Meesala, Young-Ik Sohn, Benjamin Pingault, Linbo Shao, Haig A. Atikian, Jeffrey Holzgrafe, Mustafa Gündoğan, Camille Stavrakas, Alp Sipahigil, Cleaven Chia, Ruffin Evans, Michael J. Burek, Mian Zhang, Lue Wu, Jose L. Pacheco, John Abraham, Edward Bielejec, Mikhail D. Lukin, Mete Atatüre, and Marko Lončar, "Strain engineering of the silicon-vacancy center in diamond," Physical Review B **97**, 205444 (2018).
